# Emerging Enteropathogenic *Escherichia coli* Strains?

**DOI:** 10.3201/eid1010.031093

**Published:** 2004-10

**Authors:** Tânia A.T. Gomes, Kinue Irino, Dennys M. Girão, Valéria B.C. Girão, Beatriz E.C. Guth, Tânia M.I. Vaz, Fabiana C. Moreira, Silvia H. Chinarelli, Mônica A.M. Vieira

**Affiliations:** * Universidade Federal de São Paulo, São Paulo, Brazil;; † Instituto Adolfo Lutz, São Paulo, Brazil;; ‡ Universidade de São Paulo, São Paulo, Brazil;; § Instituto Butantan, São Paulo, Brazil;; ¶ Universidade Federal de Rio de Janeiro, Rio de Janeiro, Brazil;; # Instituto Adolfo Lutz, Ribeirão Preto, São Paulo, Brazil

**Keywords:** Escherichia coli, atypical EPEC, intimin types, virulence properties, attaching and effacing, diagnosis, epidemiology, diarrhea, dispatch

## Abstract

*Escherichia coli* strains of nonenteropathogenic serogroups carrying *eae* but lacking the enteropathogenic *E. coli* adherence factor plasmid and Shiga toxin DNA probe sequences were isolated from patients (children, adults, and AIDS patients) with and without diarrhea in Brazil. Although diverse in phenotype and genotype, some strains are potentially diarrheagenic.

Typical and atypical enteropathogenic *Escherichia coli* (EPEC) strains constitute two distinct groups of organisms that have in common the locus of enterocyte effacement (LEE), a pathogenicity island that promotes the development of attaching and effacing lesions (*1**,**2*). The LEE island encompasses the *eae* gene that encodes intimin, an outer membrane adhesin fundamental to the establishment of attaching and effacing lesions (*1*). Only typical EPEC strains bear the EPEC adherence factor (EAF) plasmid, in which a cryptic sequence used as a probe (EAF probe) to the category is located (*1*).

Various evidence suggests that atypical EPEC are closer to Shiga toxin–producing *E. coli* (STEC) (*1*), which cause diarrhea and hemolytic uremic syndrome (*2*). Although many STEC strains carry LEE, their main virulence mechanism is Shiga toxin(s) (Stx) production (*2*).

Twelve EPEC serogroups (O26, O55, O86, O111, O114, O119, O125, O126, O127, O128, O142, and O158) are recognized, but recent studies have shown that most typical EPEC strains fall into only certain O:H serotypes within these serogroups, which differ from those of atypical EPEC (*1*). Furthermore, *E. coli* strains of non-EPEC serogroups that carry *eae* but lack the EAF probe sequence and *stx* genes (*eae*+ EAF– *stx– E. coli*) have been detected (*3**–**6*), but their role in endemic diarrhea has not been established, and no precise understanding of them exists. Recently, we extensively characterized a collection of such strains from a single city in Brazil (*6*). To extend our knowledge on the diversity of *eae*+ EAF *stx– E. coli* strains of non-EPEC serogroups, we compared their occurrence in three distinct cities in Brazil and their genotypic and phenotypic characteristics.

## The Study

The strains we studied were collected from patients of low socioeconomic status in three cities: São Paulo and Ribeirão Preto, in São Paulo State, and Rio de Janeiro, in Rio de Janeiro State, Brazil. The São Paulo strains were collected from 505 diarrheic and 505 nondiarrheic children (1–4 years of age) who visited Hospital Infantil Menino Jesus (April 1989–March 1990) (*7*). These strains had been previously characterized for various traits (*6*); in the present study, we tested them for new gene sequences. The Rio de Janeiro strains were collected from 372 diarrheic and 74 nondiarrheic children <5 years of age at the Instituto de Puericultura e Pediatria Martagão Gesteira, a public hospital at the Federal University of Rio de Janeiro (January 1998–December 1999, and May–December 2001). Strains from Ribeirão Preto were derived from 294 diarrheic children (<9 years of age) and adults (18–52 years), including 42 adults with AIDS. Fecal samples from these patients were sent to the Regional Laboratory of Instituto Adolfo Lutz by Hospital Santa Lydia and different clinics in the vicinity (August 2000–June 2002). This study has been approved by the Universidade Federal de São Paulo, Escola Paulista de Medicina Ethical Committee for human experimentation.

In all studies, five lactose-fermenting isolates and one nonlactose-fermenting isolate of each morphologic type, present in each fecal sample, were biochemically characterized as *E. coli*. Other well-established bacterial enteropathogens (*Salmonella* spp., *Shigella* spp., *Aeromonas* spp, *Campylobacter* spp., and *Yersinia enterocolitica*) and rotavirus were also searched for by standard methods (*8*).

All *E. coli* isolates were tested by colony hybridization with cloned or amplified genetic sequences for enterotoxigenic *E. coli*, enteroinvasive *E. coli*, EPEC (*eae* and EAF probes), STEC (*stx* probes), and enteroaggregative *E. coli*, as previously described (*6*). The *E. coli* strains that were *eae*+ EAF– *stx*– were serotyped at the Instituto Adolfo Lutz (National Reference Center for *E. coli* Serotyping) by using antisera O1 to O173 and H1 to H56.

In São Paulo and Rio de Janeiro, the *eae*+ EAF– *stx– E. coli* strains of non-EPEC serogroups occurred in similar frequencies in diarrheic and nondiarrheic children: 32 (6.3%) compared with 27 (5.3%), and 19 (5.1%) compared with 4 (5.4%), respectively. In Ribeirão Preto, such strains were found in 17 (5.8%) patients: 13 from children (1 month–9 years of age) and 4 from adults with AIDS (27–52 years of age). A total of 99 strains (one from each patient) were selected for further analysis. These strains had diverse serotypes (Table 1); 25 (25.2%) strains were nonmotile, 3 were rough, and 47 (47.5%) did not react with the O antisera tested. Among the 49 O-typable strains, 29 serogroups and 35 serotypes were found. The most frequent serotype was O51:H40 (10.1%), which occurred in all three areas studied. Most of the other serotypes occurred in one or two strains.

**Table 1:** Serotypes identified among *eae*+ EAF- *stx*- *Escherichia coli* strains outside the enteropathogenic *E. coli* serogroups^a^

Serotype (no. of strains)	Serotype (no. of strains)	Serotype (no. of strains)
O2ab:H45	O101:H33	ONT:H7 (3)
O2ab:HNT	O104:H-	ONT:H8 (4)
O4: H1	O104: H12	ONT: H9
O4: H16	O109:H9	ONT:H11
O11: H2	O115:H8	ONT:H19 (3)
O11: H16	O118:HNT (2)	ONT:H25
O13:H11	O121:H-	ONT:H29,31
O16:H-	O123:H19	ONT:H33 (3)
O19:H-	O124:H40	ONT:H34
O39:H-	O132:H8	ONT:H38
O41:H-	O145:H-	ONT:H40 (2)
O49:H10	O153:H7	ONT:H40,43 (2)
O51: H40 (10)	O154:H9	ONT:H46
O51: H-	O157:H16	ONT:HNT (3)
O63:H6 (2)	O162:H-	OR:H11,21,40
O66:H8	O162:H33	OR:H11,21
O70:H2	ONT:H- (16)	OR:H28
O85:H31 (3)	ONT:H2 (2)	
O98:H8	ONT:H6 (2)	

^a^NT, nontypable with antisera O1 to O173 and H1 to H 56; H-, nonmotile; R, rough strains.

**Figure 1:**
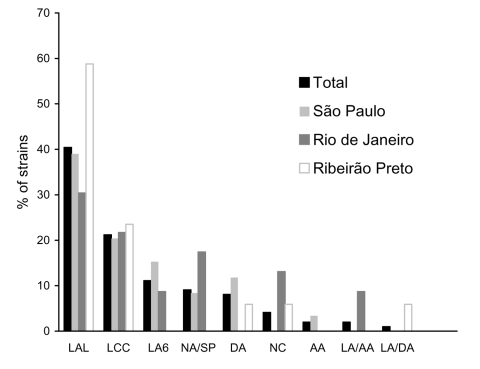
Prevalence of distinct adherence patterns in *eae*+ EAF– *stx*– *Escherichia coli* strains outside the enteropathogenic *E. coli* (EPEC) serogroups in three cities in Brazil. LAL, localized adherence-like; LCC, loose and compact clusters; LA6, localized adherence in 6-hour assay; NA/SP, nonadherent/sporadic; DA, diffuse adherence; NC, noncharacteristic; AA, aggregative adherence; LA/AA, localized and aggregative adherence; LA/DA, localized and diffuse adherence.

All strains were tested for adherence to HeLa cells (3- and 6-hour assays) (*9*). Four of them promoted sporadic adherence, four were nonadherent, and one was cytodetaching. For 88 of the 90 adherent strains, the adherence patterns could only be determined in 6 hours. Seventy-two (80.0%) of the 90 strains had variations of the localized adherence (LA) pattern of typical EPEC, which is characterized by compact bacterial clusters (*10*). These variant patterns included the following: LA-like pattern, which showed loose bacterial clusters (*11*); a pattern that showed loose and compact clusters; and a pattern identical to LA, despite its detection in only 6 hours (LA6). Other less frequent patterns included the following: the diffuse adherence typical of diffusely adhering *E. coli*, the aggregative adherence typical of enteroaggregative *E. coli* (*2*), and an association of diffuse adherence and LA or of aggregative adherence and LA. These mixed patterns were retained when individual colonies were tested. The aggregative adherence/LA pattern (two strains) was only recognized in the 3-hour assays. The prevalence of the different patterns varied by area of study, but the variations of LA were the most prevalent in all (72.7%) (Figure 1).

The ability to promote attaching and effacing lesions was tested by the fluorescent actin staining test (FAS) (*7*) in 94 strains; the 5 nonadherent or cytodetaching strains were not tested. Seventy (74.4%) of the strains tested were positive: 43 (72.9%), 15 (65.2%), and 12 (70.2%) of the strains from São Paulo, Rio de Janeiro, and Ribeirão Preto, respectively. Moreover, four distinct segments of the LEE region were found in all strains studied, as detected by hybridization with specific LEE sequences (LEE A, B, C, and D) (*12*), which suggests that all bear a complete LEE region.

LEE insertion sites were detected by a combination of polymerase chain reaction (PCR) assays with primers for the *selC* junctions and for conserved sequences of *selC* and *pheU* (*12**,**13*). LEE was inserted in *selC* in 46 strains: 24 (40.7%), 13 (56.6%), and 9 (53.0%) strains from São Paulo, Rio de Janeiro, and Ribeirão Preto, respectively. In addition, LEE was probably inserted in *pheU* in 29 (49.1%) and 3 (13.0%) of the São Paulo and Rio de Janeiro strains, respectively. In 13 strains, LEE is probably inserted in another site, since both loci were intact. The LEE insertion site was undetermined in eight strains because both *selC* and *pheU* were disrupted, and the primers for the LEE junctions in *selC* yield no amplification. Strains with an undetermined LEE insertion site occurred in all three areas studied.

Strains were also tested for 24 DNA sequences of established or putative virulence properties of pathogenic *E. coli* by colony hybridization (*6*). DNA probes were obtained from cloned genes (*bfpA*, *perA*, *E-hly*, EAEC, *daaC*, *cdt*, *cnf*, *hly*) (*6*) or by PCR amplification, which used as templates the genomic DNA of EAEC prototype strains 042 (*aafC*, *aggR*, *aspU*, *shf*, *irp2*, *pet*, and *pic*) and 17-2 (*aggC* and *astA*); extraintestinal pathogenic strains (ExPEC) J96 (*pap*) and KS52 (*afa*), and *E. coli* HB101 (pANN 801-13) (carrying the *sfa* probe). PCR primers and assay conditions used were described previously (*6**,**14*).

Hybridization with 17 of the 24 sequences tested was detected among the strains; *hly* and *irp2* (31.3% each) and *astA* (29.3%) were the most frequent. Thirty-four different combinations of these 17 sequences were found (Table 2). Their prevalence varied by location, but 25 (73.5%) occurred in two or fewer strains. Among the less frequent combinations found, some were of genes of ExPEC and EAEC, and others of genes of EPEC (*bfpA*) and EHEC (*E-hly*). Moreover, 30.3% of the strains lacked all 24 DNA sequences tested, comprising the most frequent subgroup of strains in all three areas (Table 2). Although these strains carried only the *eae* gene and the four LEE probe sequences (LEE+ only strains), they may have carried virulence sequences other than those tested. Thus, one should not emphasize the virulence potential of these LEE+ strains solely on the basis of findings of significant differences in their frequencies between cases and controls.

**Table 2:** Prevalence of distinct combinations of virulence-related DNA sequences in *eae*+ EAF- *stx*- *Escherichia coli* strains outside the EPEC serogroups in three cities in Brazil^a^

Genetic profile^b^	No. (%) of strains			
	Total (n = 99)	São Paulo (n = 59)	Rio de Janeiro (n = 23)	Ribeirão Preto (n = 17)
*eae*	31 (31.1)	19 (32.2)	5 (21.8)	7 (41.1)
*eae hly astA pet irp2*	8 (8.1)	8 (13.6)	0	0
*eae hly*	6 (6.1)	5 (8.5)	0	1(5.9)
*eae shf*	5 (5.1)	1(1.7)	3 (13.1)	1(5.9)
*eae irp2*	5(5.1)	4 (6.8)	1(4.3)	0
*eae perA bfpA astA*	4 (4.0)	1(1.7)	3 (13.1)	0
*eae perA bfpA*	4 (4.0)	0	4 (17.4)	0
*eae hly daaC afa astA pet irp2*	3 (3.0)	3 (5.1)	0	0
*eae perA*	3 (3.0)	0	0	3 (17.6)
*eae perA hly astA pet irp2*	2 (2.0)	1(1.7)	0	1(5.9)
*eae* EHEC-*hly astA*	2 (2.0)	2 (3.4)	0	0
*eae astA irp2*	2 (2.0)	2(3.4)	0	0
*eae bfpA*	2 (2.0)	1(1.7)	1(4.3)	0
*eae* EHEC-*hly*	2 (2.0)	0	2 (8.7)	0
*eae hly daaC afa pap sfa astA shf pet irp2*	1(1.0)	1(1.7)	0	0
*eae hly daaC afa shf irp2*	1(1.0)	1(1.7)	0	0
*eae perA bfpA hly pet*	1 (1.0)	0	0	1(5.9)
*eae perA hly daaC afa*	1(1.0)	0	0	1(5.9)
*eae perA bfpA astA irp2*	1(1.0)	1(1.7)	0	0
*eae hly pap afa irp2*	1(1.0)	0	1(4.3)	0
*eae hly daaC afa astA*	1(1.0)	1(1.7)	0	0
*eae hly astA shf irp2*	1(1.0)	1(1.7)	0	0
*eae perA bfpA hly*	1(1.0)	0	0	1(5.9)
*eae hly astA irp2*	1(1.0)	1(1.7)	0	0
*eae hly shf irp2*	1(1.0)	1(1.7)	0	0
*eae perA astA*	1(1.0)	0	1(4.3)	0
*eae* EHEC-*hly bfpA*	1(1.0)	1(1.7)	0	0
*eae hly shf*	1(1.0)	1(1.7)	0	0
*eae hly irp2*	1(1.0)	1(1.7)	0	0
*eae hly astA*	1(1.0)	1(1.7)	0	0
*eae astA shf*	1(1.0)	0	0	1(5.9)
*eae shf irp2*	1(1.0)	0	1(4.3)	0
*eae astA*	1(1.0)	1(1.7)	0	0
*eae cdt*	1(1.0)	0	1(4.3)	0

^a^EPEC, enteropathogenic *Escherichia coli*; EHEC, enterohemorrhagic *E. coli*. ^b^All strains hybridized with the locus of enterocyte effacement (LEE) A, LEE B, LEE C, and LEE D probes constructed by McDaniel et al. (*12*), which suggested that they bear a complete LEE region.

DNA sequences similar to *bfpA* were detected in 14 (14.1%) of the 99 strains studied, however, only 2 expressed Bfp in Western blot experiments (not shown); these two strains also carried *perA* and presented AA/LA in 3 hours. The HeLa pattern of the remaining *bfpA*+ strains varied, but none of them had compact clusters in 3 hours, which is typical of LA. Thus, Bfp expression was found only in strains presenting aggregative adherence/LA in 3 hours, as in typical LA of EPEC (*1*).

**Figure 2:**
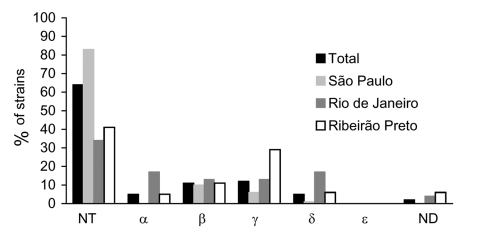
Intimin types in *eae*+ EAF– *stx*– *Escherichia coli* strains outside the enteropathogenic *E. coli* (EPEC) serogroups in three cities in Brazil. NT, nontypable with the sequences tested; ND, not done.

PCR assays with specific primers for the variable region of intimin were used to identify five intimin types (α, β, γ, δ, and ε) (*15**,**16*). Most strains had a nontypable intimin (64.6%), but the distribution of these strains varied (approximately 70% in São Paulo and 29%–35% in Rio de Janeiro and Ribeirão Preto). Recently, new schemes were proposed to identify intimin subtypes, which were not tested (*17**,**18*). The prevalence of typable intimins varied among the three areas analyzed. Intimin subtypes β (11.1%) and γ (12.1%) prevailed, and intimin ε was not found (Figure 2). The intimin types of two strains were not determined because amplification products of the expected size were obtained with four intimin pairs of primers.

## Conclusions

In this study, we sought to verify the frequency with which *eae*+ EAF– *stx– E. coli* strains of non-EPEC serogroups occur in persons of poor socioeconomic status in three Brazilian cities; we also compared these strains' genotypic and phenotypic characteristics. Although these strains occurred in 5% to 6% of the populations studied, including nondiarrheic children (in São Paulo and Ribeirão Preto), 73%–88 % of them were dissociated from other well-established enteropathogens (not shown).

Although O51:H40 was the most frequent serotype found and occurred in all three areas studied, the non-EPEC *eae*+ EAF– *stx*– strains comprised a large variety of serotypes, and many were O nontypable. Moreover, the strains had diverse adherence patterns and various combinations of pathogenic *E. coli* DNA virulence sequences; the prevalence of these properties varied among the areas studied. Altogether, these data show that *eae*+ EAF– *stx– E. coli* strains outside the EPEC serogroups are even more diverse than already observed (*6*). As we have emphasized previously, such diversity challenges the diagnosis of these putative pathogens (*6*).

All strains carried an apparently complete LEE region, and approximately 75.0% of them had the potential to promote attaching and effacing lesions in HeLa cells, as detected by FAS. Thus at least these FAS+ strains are potentially enteropathogenic, since they are capable of inducing attaching and effacing lesions in vitro and may occur in diarrheic patients of various ages and in patients with AIDS. In the EPEC meeting held in 1995, a consensus definition of atypical EPEC was established, namely, that they are EAF–, *eae*+ strains that promote attaching and effacing lesions (*19*). Therefore, the FAS+ strains of our study could be classified as atypical EPEC. Whether these strains have additional virulence properties not present in typical EPEC remains to be elucidated. Studies on the virulence potential of selected strains at the cellular and molecular levels will certainly contribute to further understanding of this group of strains and aid in discriminating enteropathogenic strains within the group.

## References

[1] Trabulsi LR, Keller R, Gomes TAT. Typical and atypical enteropathogenic *Escherichia coli* (EPEC). Emerg Infect Dis. 2002;8:508–13. 11996687 10.3201/eid0805.010385 PMC2732489

[2] Nataro JP, Kaper JB. Diarrheagenic *Escherichia coli.* [Erratum in Clin Microbiol Rev. 1998;11:403]. Clin Microbiol Rev. 1998;11:142–201. 9457432 10.1128/cmr.11.1.142 PMC121379

[3] Afset JE, Bergh K, Bevanger L. High prevalence of atypical enteropathogenic *Escherichia coli* (EPEC) in Norwegian children with diarrhea. J Med Microbiol. 2003;52:1015–9. 10.1099/jmm.0.05287-0 14532347

[4] Dulguer MV, Fabbricotti SH, Bando SY, Moreira-Filho CA, Fagundes-Neto U, Scaletsky ICA. Atypical enteropathogenic *Escherichia coli* strains: phenotypic and genotypic profiling reveals a strong association between enteroaggregative *E. coli* heat-stable enterotoxin and diarrhea. J Infect Dis. 2003;188:1685–94. 10.1086/379666 14639540

[5] Regua-Mangia AH, Gomes TAT, Vieira MAM, Andrade JCR, Irino K, Teixeira LM. Frequency and characteristics of diarrheagenic *Escherichia coli* strains isolated from children with and without diarrhea in Rio de Janeiro, Brazil. J Infect. 2004;48:161–7. 10.1016/S0163-4453(03)00138-5 14720492

[6] Vieira MAM, Andrade JRC, Trabulsi LR, Rosa ACP, Dias AMG, Ramos SRTS, Phenotypic and genotypic characteristics of *Escherichia coli* strains of non-enteropathogenic *E. coli* (EPEC) serogroups that carry *eae* and lack the EPEC adherence factor and Shiga toxin DNA probe sequences. J Infect Dis. 2001;183:762–72. 10.1086/318821 11181153

[7] Gomes TAT, Griffin PM, Ivey C, Trabulsi LR, Ramos SRTS. EPEC Infections in São Paulo. Rev Microbiol, São Paulo. 1996;27:25–33.

[8] Cravioto A, Gross RJ, Scotland SM, Rowe B. An adhesive factor found in strains of *Escherichia coli* belonging to the traditional infantile enteropathogenic serotypes. Curr Microbiol. 1979;3:95–9. 10.1007/BF02602439

[9] Scaletsky ICA, Silva MLM, Trabulsi LR. Distinctive patterns of adherence of enteropathogenic *Escherichia coli* to HeLa cells. Infect Immun. 1984;45:534–6. 6146569 10.1128/iai.45.2.534-536.1984 PMC263286

[10] Rodrigues J, Scaletsky ICA, Campos LC, Gomes TAT, Whittam ST, Trabulsi LR. Clonal structure and virulence factors in strains of *Escherichia coli* of the classic serogroup O55. Infect Immun. 1996;64:2680–6. 8698495 10.1128/iai.64.7.2680-2686.1996 PMC174126

[11] Knutton S, Baldwin T, Williams PH, McNeish AS. Actin accumulation at sites of bacterial adhesion to tissue culture cells: basis of a new diagnostic test for enteropathogenic and enterohemorrhagic *Escherichia coli.* Infect Immun. 1989;57:1290–13. 2647635 10.1128/iai.57.4.1290-1298.1989 PMC313264

[12] McDaniel TK, Jarvis KG, Donnenberg MS, Kaper JB. A genetic locus of enterocyte effacement conserved among diverse enterobacterial pathogens. Proc Natl Acad Sci U S A. 1995;92:1664–8. 10.1073/pnas.92.5.1664 7878036 PMC42580

[13] Sperandio V, Kaper JB, Bortolini MR, Neves BC, Keller R, Trabulsi LR. Characterization of the locus of enterocyte effacement (LEE) in different enteropathogenic *Escherichia coli* (EPEC) and Shiga-toxin producing *Escherichia coli* (STEC) serotypes. FEMS Microbiol Lett. 1998;164:133–9. 10.1111/j.1574-6968.1998.tb13078.x 9675859

[14] Elias WP, Uber AP, Tomita S, Trabulsi LR, Gomes TAT. Combinations of putative virulence markers in typical and variant enteroaggregative *Escherichia coli* strains from children with and without diarrhea. Epidemiol Infect. 2002;129:49–55. 10.1017/S0950268802007136 12211596 PMC2869874

[15] Adu-Bobie J, Frankel G, Bain C, Gonçalves AG, Trabulsi LR, Douce G, Detection of intimin alpha, beta, gamma, and delta, four intimin derivatives by attaching and effacing microbial pathogens. J Clin Microbiol. 1998;36:662–8. 9508292 10.1128/jcm.36.3.662-668.1998 PMC104605

[16] Oswald E, Schmidt H, Morabito S, Karch H, Marchès O, Caprioli A. Typing of intimin genes in human and animal enterohemorrhagic *Escherichia coli*: characterization of a new intimin variant. Infect Immun. 2000;68:64–71. 10.1128/IAI.68.1.64-71.2000 10603369 PMC97102

[17] Jenkins C, Lawson AJ, Cheasty T, Willshaw GA, Wright P, Dougan G, Subtyping intimin genes from enteropathogenic *Escherichia coli* associated with outbreaks and sporadic cases in the United Kingdom and Eire. Mol Cell Probes. 2003;17:149–56. 10.1016/S0890-8508(03)00046-X 12944116

[18] Zhang WL, Köhler B, Oswald E, Beutin L, Karch H, Morabito S, Genetic diversity of intimin genes of attaching and effacing *Escherichia coli* strains. J Clin Microbiol. 2002;40:4486–92. 10.1128/JCM.40.12.4486-4492.2002 12454140 PMC154638

[19] Kaper JB. Defining EPEC. Proceedings of the International Symposium on Enteropathogenic *Escherichia coli* (EPEC). Rev Microbiol São Paulo. 1996;27:130–3.

